# **PROTOCOL**: Examining the best time of day for exercise: A systematic review and network meta‐analysis

**DOI:** 10.1002/cl2.1144

**Published:** 2021-06-21

**Authors:** Meixuan Li, Xiuxia Li, Liujiao Cao, Rui Li, Xiaoqin Wang, Liang Yao, Peijing Yan, Yanfei Li, Xiajing Chu, Huijuan Li, Xue Han, Tianjiao Xin, Kaiyue Chen, Howard White, Kehu Yang

**Affiliations:** ^1^ Evidence‐Based Medicine Center, School of Basic Medical Sciences Lanzhou University Lanzhou China; ^2^ Evidence‐Based Social Science Research Center, School of Public Health Lanzhou University Lanzhou China; ^3^ The Michael G. DeGroote Institute for Pain Research and Care McMaster University Hamilton Canada; ^4^ Department of Health Research Methodology, Evidence and Impact McMaster University Hamilton Canada; ^5^ Department of Clinical Research Management, West China Hospital Sichuan University Chengdu China; ^6^ The First School of Clinical Medicine Lanzhou University Lanzhou China; ^7^ Campbell Collaboration New Delhi India

## 1 BACKGROUND

### 1.1 The problem, condition or issue

Global health is influenced by factors such as ageing population, rapid urbanisation, and increased transportation by car and plane, all of which result in unhealthy environments and behaviour. As a result, the growing prevalence of noncommunicable diseases (NCDs) and their risk factors has become a global issue (Zhang et al., 2017). According to the WHO World Health Statistics 2018 (WHO, 2018), In 2016, an estimated 41 million deaths occurred due to NCDs, accounting for 71% of the overall total of 57 million deaths. The majority of such deaths were caused by the four main NCDs, including: cardiovascular disease (17.9 million deaths; accounting for 44% of all NCD deaths); cancer (9.0 million deaths; 22%); chronic respiratory disease (3.8 million deaths; 9%); and diabetes (1.6 million deaths; 4%).

There are many key risk factors of NCDs such as tobacco use, air pollution, unhealthy diet, physical inactivity and harmful use of alcohol, one of the main risk factors is physical inactivity (WHO 2018). Physical inactivity has been identified as the fourth leading risk factor for global mortality (6% of deaths globally), with an estimated 20%–30% increased risk of death compared with those who are physically active. This follows high blood pressure (13%), tobacco use (9%) and high blood glucose (6%). Overweight and obesity are responsible for 5% of global mortality (WHO, 2013). It has been shown that participation in regular physical activity reduces the risk of coronary heart disease and stroke, diabetes, hypertension, colon cancer, breast cancer and depression. Additionally, physical activity is a key determinant of energy expenditure, and thus is fundamental to energy balance and weight control (WHO, 2002, 2005, 2007, 2008, 2010).

Physical activity is defifined as bodily movement produced by skeletal muscle contraction that requires energy expenditure above basal levels. It includes activities related to activities of daily life, such as housekeeping, yardwork, occupational‐related, leisure‐related and transportation. Exercise typically is differentiated from physical activity in that it is typically planned, repetitive, and structured with the main objective of improving health and fifitness. Physical fifitness is a state of good health and strength achieved through physical activity and exercise (Fletcher et al., 2018). The main types of physical exercise include aerobic exercise, resistance training (anaerobic exercise), flexibility and balance. Aerobic exercise increases the uptake of oxygen in the larger muscle groups and has a beneficial effect on cardiovascular homoeostasis. Resistance training mainly affects muscle strength and mass whereas flexibility and balance exercise improve the range of motion necessary for daily activity and diminish the risk of falls (Nehrlich, 2006; Nelson et al., 2007).

Being physically active and exercise is one of the most important actions individuals of all ages can engage in to improve their health. The evidence reviewed by the Physical Activity Guidelines Advisory Committee 4 for the newly released Physical Activity Guidelines for Americans, 2nd edition 5 (PAG) is clear—physical activity fosters normal growth and development and can make people feel better, function better, sleep better, improve wellbeing and reduce the risk of many chronic diseases (FUüzeki et al., 2017; Hills et al., 2015; Nehrlich, 2006; Warburton Darren & Bredin Shannon, 2017).

However, many factors may influence the benefits of doing regular exercise, such as the exercise type, duration, hormone adaptation, and timing of exercise (Seo et al., 2013). To maximise the benefits of exercise, more tailored exercise prescriptions are required. Abundant scientific evidence has demonstrated that time of day is one of the important factors that affects the outcomes related to exercise. However, there remains much debate when to exercise and when to do what type of exercise. Is exercising in the evening, mid‐day or in the morning is better for human health?

### 1.2 The intervention

#### 1.2.1 How the intervention might work

The existence of a time‐of‐day effect on human performance is now well established. However, the optimal timing of exercise for health have not been fully established (Kraemer et al., 2001).

**Figure 1:**
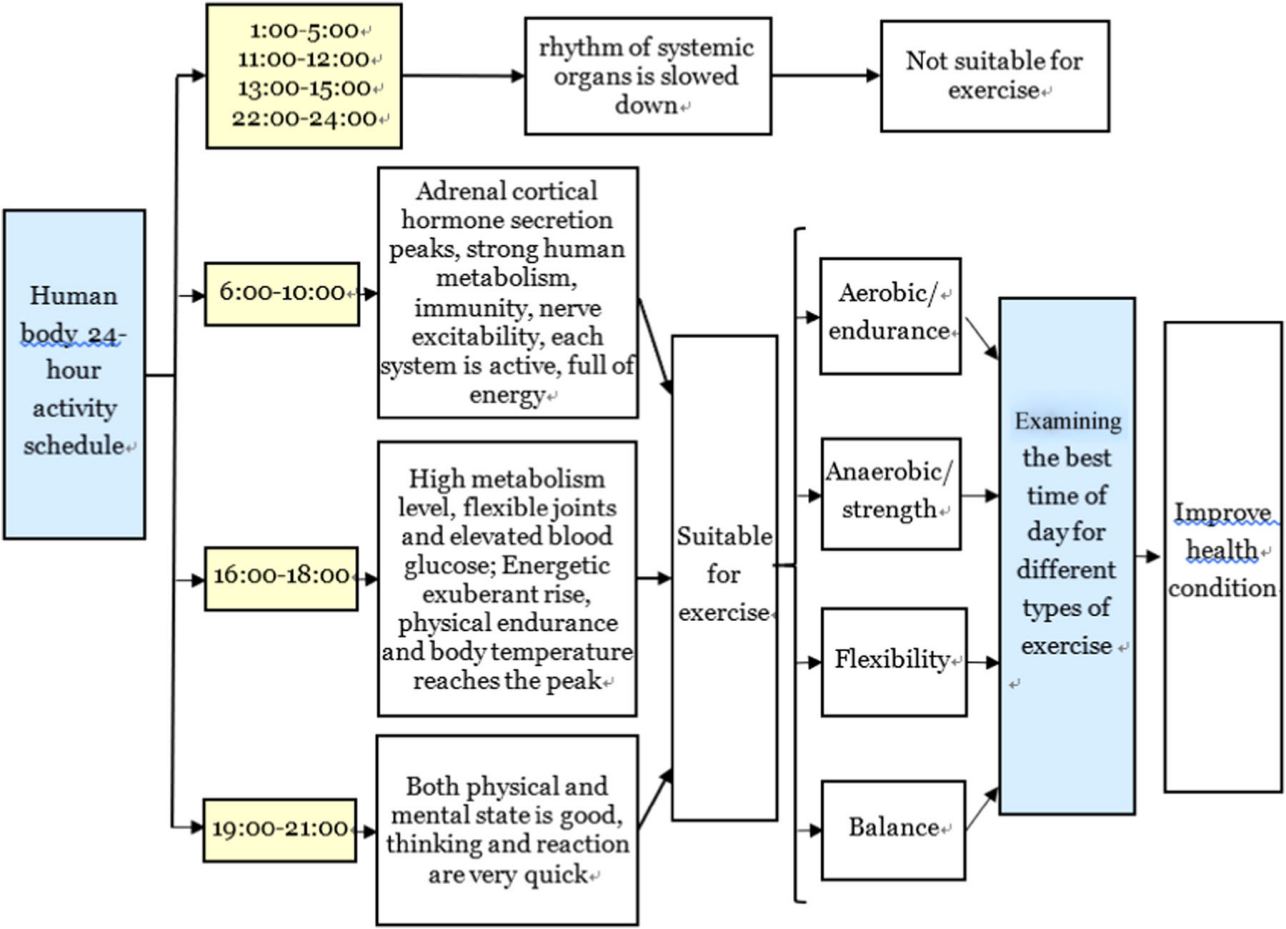
Human body 24‐h activity change rhythm

Numerous physiological phenomena in the human body, such as sleep–wake cycles, hormonal and nervous activity, and body temperature, exhibit rhythmic changes over the course of 24 h (Bass & Takahashi, 2010; Yu & Shibata, 2013). It is already known that hormone concentrations exhibit circadian rhythmicity and so vary throughout the day (Kraemer et al., 2001) along with body temperature (Bailey & Heitkemper, 2001) and strength performance (Sedliak et al., 2009). With the different hormone concentrations, motor function (such as strength, whole‐body flexibility, simple reaction time and short‐term power output) display a time‐of‐day effect characterised by a late afternoon acrophase (~18:00 h) and an early morning bathyphase (~06:00 h; see reviews by Atkinson & Reilly, 1996, Reilly et al., 2000). More specific changes of body indicators in 24 h are shown in Figure 1, we can see in the morning (06:00–10:00 h), afternoon (16:00–18:00 h), and evening(19:00–21:00 h), our body is the most energetic and suitable for exercise, which needs to take a lot of energy and strength. However, research on optimising exercise timing is sparse. In other words, which is the best time of the day (morning, afternoon and evening) to trigger an optimal training response remains unclear (Gabriel & Zierath, 2019).

#### 1.2.2 Why it is important to do this review

*The Physical Activity Guidelines for Americans*, 2nd edition (Piercy et al., 2018), provides information and guidance on the types and amounts of physical activity that provide substantial health benefits. But it has no recommendation on the best time of a day to exercise. There are several RCTs (Brito et al., 2018; Drust et al., 2005; Gabriel & Zierath, 2019; Souissi et al., 2002) focusing on the timing of exercise. One RCT with 10,086 participants which indicated that body weight, body mass index, abdominal skin fold thickness and abdominal circumference decreased significantly more with morning exercise compared to evening exercise over a 6 week period (Gabriel & Zierath, 2019). Souissi et al (Souissi et al., 2002) suggested that several weeks of repeated strength training performed in the morning hours may reduce the typical diurnal pattern by increasing maximum strength more in the morning than at other times of day. However, the results about different timing for exercise are not always consistent. For example, Drust et al. (2005) reported that maximum voluntary strength of untrained men typically exhibits a diurnal pattern, with low morning values and peak values in the afternoon. And it's worth noting that the effect of time on exercise effects may vary by age, as people of different ages have different physical qualities.

Knowing the right time to do exercise could be valuable for people to improve the quality of life, but the conflicting results from current evidence make it difficult to draw a conclusion to this question. Therefore, it is necessary to systematically gather and synthesise published and unpublished evidence to examine the best time of day for different types exercise.

## 2 OBJECTIVES

The primary objective of the review is to synthesise evidence on the effectiveness of exercise in different times of a day. The review aims to answer the following question:

Which is the best time of day for different types of exercise—morning, afternoon or evening—to obtain the highest benefits such as physical and mental health, weight loss and so on?

## 3 METHODS

### 3.1 Criteria for considering studies for this review

#### 3.1.1 Types of studies

The proposed review will include following three types of studies: (1) randomised controlled trials in which participants are randomly assigned to an experimental or control group, (2) quasi‐randomised controlled trials in which participants are allocated by means such as alternate allocation, person's birth date, the date of the week or month, or alphabetical order, and (3) nonrandomised controlled trials in which participants are nonrandomly assigned to an experimental or control group.

In these designs, studies compared exercise during different times of day, or studies that compared exercise during a specific time of day with no exercise, for example, a study compared exercise in the morning to exercise in the afternoon; or studies that compared exercise in the morning with no exercise, the experimental group refers to participants who exercise in the morning, afternoon, or evening to improve their health, and the control group refers to those in no‐training. We will also include studies comparing different types of exercise at the same or different specific time of day.

#### 3.1.2 Types of participants

The review will include all human populations including any age, sex and health status.

#### 3.1.3 Types of interventions

Studies will be included if their interventions meet the following criteria:

(1) included exercise, that is physical activity comprising planned, structured and repetitive body movements, which are undertaken to improve one or more components of physical fitness according to the American College of Sports Medicine (Ferguson, 2014);

(2) compared exercise during different times of day, or studies that compared exercise during a specific time of day versus no exercise.

#### 3.1.4 Types of outcome measures

##### Primary outcomes

The primary outcome measures include physical health, mental health, general health/ill health and quality of life indicators measured using validated instruments (some possible examples are listed in Table 1).

**Table 1:** Examples of primary outcomes measured

Primary outcomes	Examples of measurements
Physical health, for example, musculoskeletal disorders, strength performance. Chronic diseases (hypertension, diabetes, cardiovascular diseases, etc.), fatigue, appetite	Physician diagnoses: McGill Pain Questionnaire (MPQ), change in blood pressure, body mass index (BMI) or other physiological parameters
Mental health, for example, depression, anxiety	General Health Questionnaire (GHQ‐12) RAND Mental Health Inventory (MHI) Hospital Anxiety and Depression Scale (HADS) Warwick‐Edinburgh Mental Well‐being Scale
General health/ill health	UK Census style measures of general health and limiting long‐term illness
Quality of life	EORTC (European Organisation for Research and Treatment of Cancer) Quality of Life Questionnaire Functional Limitations Profile (FLP) Short Form (SF‐36) EuroQol (EQ‐5D)
Sleep quality	Scores on the PGI‐C

##### Secondary outcomes

We will extract outcomes relating to social wellbeing,specifically work‐life balance, but only when reported alongside primary outcomes and when work‐life balance was measured using a validated instrument. We will also extract some anthropometric indices, such as heart rate, calorie intake, macronutrient consumption, weight loss and so on, shown in Table 2.

**Table 2:** Examples of secondary outcomes measured

Secondary outcomes	Possible examples of measurements
Work‐life balance	Social/domestic disruption/interference Family‐to‐work conflict Time spent with friends/family Time spent on domestic chores/hobbies
Anthropometric indices	For example, heart rate, calorie intake, macronutrient consumption, weight loss

###### Duration of follow‐up

Studies with any length of follow up will be included in this review. In order to synthesise data from studies with different lengths of follow‐up, groups will be defined and analysed separately (eg., 0–6, 6–12 and >12 weeks) and will be pooled where there are no significant differences.

###### Types of settings

Studies that report participants in any setting will be included.

### 3.2 Search methods for identification of studies

We will search electronic databases, grey literature sources and hand search journals to identify all potentially eligible studies. There will be no restrictions placed on document language or publication status. We will also contact leading authors and experts in the field of exercise and health for additional studies via email. The bibliographies of relevant reviews and included studies will be hand searched to identify additional references for review. The search strategy of Medline is as follows:

"Exercise"[Mesh]) OR Exercise*[Title/Abstract] OR Physical Activit*[Title/Abstract]) OR Training[Title/Abstract]

AND

"Time"[Mesh] OR Time[Title/Abstract] OR timing[Title/Abstract] OR Morning[Title/Abstract] OR Afternoon[Title/Abstract] OR Evening[Title/Abstract] OR schedule[Title/Abstract] OR night[Title/Abstract] OR noon[Title/Abstract]

#### 3.2.1 Electronic searches

Medline

Embase

Web of Science

Cochrane Central Register of Controlled Trials (CENTRAL)

Chinese Biomedical Literature Database (CBM)

China Network Knowledge Information (CNKI)

Wanfang Database

Social Care Online: https://www.scie-socialcareonline.org.uk/.

AgeLine: http://www.aarp.org/research/ageline/.

Global Health: http://www.cabi.org/datapage.asp?iDocID=169.

Database of Promoting Health Effectiveness Reviews (DoPHER): http://eppi.ioe.ac.uk/webdatabases/Intro.aspx?ID=2.

#### 3.2.2 Searching other resources

##### Grey literature sources

We will search Google Scholar, OpenSIGLE (http://opensigle.inist.fr/), Opengrey (http://www.opengrey.eu/), Clinicaltrials.gov (https://www.clinicaltrials.gov/), World Health Organization International Clinical Trials Registry Platform (https://www.who.int/ictrp/en), Social Science Research Network (https://www.ssrn.com/index.cfm/en/) and major sports science institutions (https://www.lboro.ac.uk/departments/ssehs/; https://www.acsm.org/) for grey literature. Copies of relevant documents will be made and we will record the exact URL and date of access for each relevant document.

###### Hand searching

In addition to check the reference lists of included studies and reference lists of relevant reviews, we will also hand search following relevant journals:

Medicine and science in sports and exercise

Psychology of sport and exercise

Research quarterly for exercise and sport

Scimago Institutions Rankings (https://www.scimagojr.com/journalrank.php?Category=3699), which included many journals of sports, such as British Journal of Sports Medicine, American Journal of Sports Medicine and so on.

### 3.3 Data collection and analysis

#### 3.3.1 Selection of studies

Endnote X9 software will be used to manage retrieved bibliographies. EPPI‐Reviewer 4 will be used to screen retrieved bibliographies and extract data. After the removal of duplicate results, two reviewers will first independently screen titles and abstracts to exclude studies that are clearly irrelevant. If studies are considered eligible by at least one assistant or there is insufficient information in the title and abstract to judge eligibility, will be retrieved in full text. The selected review author pair will collect full‐text trial publications, and independently screen the full‐texts and identify trials for inclusion, any disagreement of eligibility will be resolved by a third party from the review authors. Exclusion reasons for studies that otherwise might be expected to be eligible will be documented and listed in an appendix. We will record the selection process in sufficient detail to complete a PRISMA flow diagram and ‘Characteristics of excluded studies’ table for studies excluded on full text.

#### 3.3.2 Data extraction and management

Two reviewers, working in pairs, will independently extract data using data extraction forms designed for the purpose, and we will pilot the form against sample studies before finalising. If there is disagreement, the authors will discuss the reasoning behind their assessment. If an agreement is not reached between the two authors, YL will serve as arbitrator. The following information will be extracted from each included study:

1. Document description (e.g., first author, year of publication, country)
2. Methodological issues (e.g., design, randomisation, blinding, selective reporting)
3. Participant information (e.g., age, gender, health status, muscle mass, fat mass, body fat)
4. Intervention (e.g., exercise time, exercise type, exercise duration)
5. Outcomes (e.g., wellbeing, sleep quality, weight loss, heart rate)
6. The length of follow up

#### 3.3.3 Assessment of risk of bias in included studies

Two authors will independently assess the risk of bias in each included study. Discrepancies will be discussed with a third author until consensus is achieved. Randomised controlled trials and quasi‐randomised controlled trial will be assessed using the tool recommended by the *Cochrane Handbook Version 5.1.0* (Higgins & Green, 2011), We will assess the following domains: risk of bias, allocation sequence generation, allocation concealment, blinding of outcome assessors, incomplete outcome data, selective outcome reporting and other potential sources of bias (i.e., the length of training and confounding variables). RCTs will not be assessed for blinding of participants as the participatory nature of interventions (exercise in different time) makes blinding impossible. When the risk of bias of all seven terms is defined as “low risk of bias”, the trial will be defined as the overall “low risk of bias”. At the same time, when one or more of the seven bias components are classified as high risk, the trial will be graded as “High risk of bias”. In other cases, the trial will be graded “Unclear risk”.

Non‐RCTs will be evaluated according to ROBINS‐I (“Risk Of Bias In Non‐randomised Studies of Interventions”) (Sterne et al., 2016), which is a new tool for evaluating risk of bias in estimates of the comparative effectiveness (harm or benefit) of interventions from studies that did not use randomisation to allocate units (individuals or clusters of individuals) to comparison groups, such as cohort studies and case‐control studies in which intervention groups are allocated during the course of usual treatment decisions, and quasi‐randomised studies in which the method of allocation falls short of full randomisation. ROBINS‐I includes seven domains, the first two domains, covering confounding and selection of participants into the study, address issues before the start of the interventions that are to be compared (“baseline”). The third domain addresses classification of the interventions themselves. The other four domains address issues after the start of interventions: biases due to deviations from intended interventions, missing data, measurement of outcomes and selection of the reported results.

#### 3.3.4 Measures of treatment effect

For dichotomous outcomes, such as number of patients with chronic diseases, we will estimate the odds ratio (OR) and its 95% confidence interval (CI) using the Mantel Haenszel random‐effects model. When utilising the Mantel Haenszel method for dichotomous outcomes of rare events, we will perform an correction in case of treatment arms with zero events, which will be considered to measure a potential effect and a value of 0.5 will be added to studies that reported zero event.

**Figure 2:**
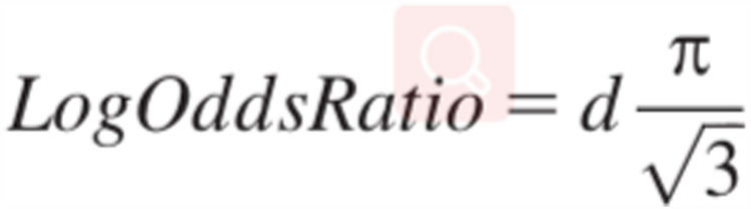
The formula for converting a LogOddsRatio to the standardised mean difference

For continuous outcomes, such as depression, anxiety, quality of life and sleep quality, the included studies may use varied rating scales for the same outcome, standardised mean diferences (SMDs) with 95% CIs will be calculated to synthesise the effects, assuming that they are normally distributed. If the units of the same outcome measure are consistent for different studies, mean difference with their respective 95% CIs will be calculated to synthesise the effects. If studies did not report standard difference (SDs), SDs will be calculated from *SE* and 95% CI using methods proposed by the Cochrane Handbook (Higgins & Green, 2011). If a mix of OR and SMD was reported for one outcome, we will convert the SMD to logOR using the formula in Figure 2 reported by Borenstein et al. (2021), and then pool them.

#### 3.3.5 Unit of analysis issues

For trials with more than two arms, we will split the “shared” group into two or more groups with smaller sample size and include two or more (reasonably independent) comparisons (Deeks et al., 2020). For example, in a trial with three arms A, B and C, where the comparisons of interest are the number of outcome events in A versus B and B versus C, we will divide the sample size of B by two and round it to the nearest whole number, and likewise the number of outcome events. We will use this smaller group in the comparisons against A and C (Lunny et al., 2020).

#### 3.3.6 Dealing with missing data

We will identify incomplete outcome data during data extraction. Where the trial report suggests that outcome data were available but not reported, we will contact the corresponding author to request missing data. Where a trial has been registered and a relevant outcome was specified in the trial protocol but no results reported, we will contact authors and funders to request trial reports

#### 3.3.7 Assessment of heterogeneity

We will explore the methodological and clinical heterogeneity of the included studies by comparing participants' characteristics (age, gender, race and the health status), interventions (the type of exercise, the time of follow up, duration of exercise), using information reported in the “Characteristics of included studies” table.

In pairwise meta‐analyses, we will examine statistical heterogeneity visually using forest plots. Statistical heterogeneity will also be assessed through Cochran *Q* test and *I* ^2^ tests. Higgins’ *I* ^2^ statistic <25%, 25%–50%, and >50% was considered indicative of low, moderate, and high heterogeneity, respectively (Higgins & Thompson, 2002). When the *I* ^2^ statistic value is greater than 50% (substantial heterogeneity), we will perform subgroup analysis and meta‐regression to consider possible reasons for heterogeneity.

##### Assessment of inconsistency

In network meta‐analysis, inconsistency can be considered an additional layer of heterogeneity which can occurin networks of evidence. It can occur when there is a discrepancy between a direct and indirect estimate of treatment effect (Guaiana et al., 2020).We will use the node splitting method to generate the effect size and credible intervals for the indirect comparison and for the statistical test of inconsistency between direct and indirect estimates (van Valkenhoef et al., 2016). We will conduct these analyses on the primary outcomes.

#### 3.3.8 Assessment of reporting biases

If 10 or more studies are included in an analysis, we will use funnel plots to assess publication bias at the study level. Selective outcome reporting will be assessed as part of the domain‐based assessment of the risk of bias in the included studies.

#### 3.3.9 Data synthesis

1. Pairwise meta‐analysis
  Where at least two studies are available, we will perform pairwise meta‐analyses for all outcomes and comparisons (all contrasts with data from two or more studies). We will assess clinical heterogeneity by comparing the PICO characteristics across studies, and will not combine clinically dissimilar groups in a meta‐analysis. We will conduct meta‐analyses in Review Manager (RevMan Web, 2019), using the DerSimonian and Laird (1986) random effects model. We will analyse all randomised participants according to the intention‐to‐treat principle, with the assumption that participants who were lost to follow‐up did not respond to treatment. We will present in figures and tables the number of participants, the summary effect measures for each comparison (i.e., mean and *SD* for each treatment class), and the OR with its 95% CIs.
2. Network meta‐analysis
  A network meta‐analysis within a frequentist model will be used to combine direct and indirect evidence from all available trials, and the mvmeta package basing on a multiple regression of the Stata software (Stata Corporation) will be used to process the network meta‐analysis. The function of “network plot” of Stata softwarewill be used to generate network plots to describe and present the geometry of different times of exercise. The nodes will be used to represent different times and edges to represent the head‐to‐head comparisons between times. Results will be reported with 95%CIs, and a *p *< .05 will be considered statistically significant. A random effects model will be used to calculate pooled estimates and 95% CI because it takes into account the almost inevitable natural variation inherent between studies, especially great use for conducting network meta‐analysis.

##### Ranking probabilities

For primary outcomes, we will display the ranking probabilities of interventions by the surface under the cumulative ranking curve (SUCRA) (Veroniki et al., 2016) which would show the probability that one intervention is better than other interventions. We will create a two‐dimensional plot for ranking with SUCRA values for acceptability in the *x* axis and SUCRA values for efficacy in the *y* axis (Chaimani et al., 2013). Studies grouped in the upper right hand quadrant will be considered to have the best balance of acceptability and efficacy.

#### 3.3.10 Subgroup analysis and investigation of heterogeneity

If we detect heterogeneity, we will apply pairwise meta‐regression models and do subgroup analyses; likewise, if we find inconsistency in network meta‐analysis models, we will use network meta‐regression. Meta‐regression will be used as the overall analysis of moderator effects. This technique reduces the probability of type I error by computing concurrent estimates of independent effects by multiple moderators on the variation in effect size across trials. We will systematically examine all possible moderators as following, which may influence the effects of exercise.

a) Variation in programme participants (e.g., gender, age, race);
b) The time of follow up (e.g., 0–6, 6–12 and >12 weeks);
c) The health status (e.g., cancer patients, overweight patients, chronic disease patients, healthy population);
d) The type of exercise (e.g., aerobic activity, balance activities).

#### 3.3.11 Sensitivity analysis

We plan to conduct sensitivity analyses on trials classified as having high quality versus trials classified as low quality; or RCTs versus non‐RCTs. We will assess the impact of any study that has a large effect size on the results of the meta‐analysis.

##### Summary of findings and assessment of the certainty of the evidence

We will use GRADE (Grades of Recommendation, Assessment, Development and Evaluation) system (Guyatt et al., 2011) to assess the certainty of evidence associated with specific outcomes and construct a “Summary of findings” table. The GRADE approach is used to assess the quality of a body of evidence based on the extent to which one can be confident that an estimate of effect or association reflects the item being assessed. Assessment of the quality of evidence considers risk of bias, inconsistency, indirectness, imprecision, publication bias and other bias (Norris et al., 2016).

##### Treatment of qualitative research

We do not plan to include qualitative research.

## CONTRIBUTIONS OF AUTHORS

- *Content*: Meixuan Li, Xiuxia Li, Liujiao Cao, Rui Li, Xiaoqin Wang and Liang Yao.
- *Systematic review methods*: Xiuxia Li, Liang Yao, Howard White and Kehu Yang.
- *Statistical analysis*: Peijing Yan,Meixuan Li, Liujiao Cao and Rui Li.
- *Information retrieval*: Yanfei Li, Xiajing Chu, Huijuan Li, Xue Han, Tianjiao Xin, Kaiyue Chen and Meixuan Li.

## DECLARATIONS OF INTEREST

Howard White is the Chief Executive Officer of the Campbell Collaboration, other authors have no conflicts of interest.

### PRELIMINARY TIMEFRAME

Approximate date for submission of the systematic review: January 2021.

### PLANS FOR UPDATING THIS REVIEW

Meixuan Li, Xiuxia Li, Liujiao Cao, Rui Li, Yanfei Li, Liang Yao and Kehu Yang will be responsible for updating the system evaluation every 5 years.
